# Usability of the Experience Sampling Method in Parkinson's Disease on a Group and Individual Level

**DOI:** 10.1002/mds.28028

**Published:** 2020-05-30

**Authors:** Anne E.P. Mulders, Rachel M.J. van der Velden, Marjan Drukker, Martijn P.G. Broen, Mark L. Kuijf, Albert F.G. Leentjens

**Affiliations:** ^1^ Department of Psychiatry and Neuropsychology School for Mental Health and Neuroscience, MHeNS, Maastricht University Maastricht the Netherlands; ^2^ Department of Neurology Maastricht University Medical Center Maastricht the Netherlands

**Keywords:** Parkinson's disease, experience sampling, motor fluctuations, affective symptoms, methodological issues

## Abstract

**Background:**

Around 50% of PD patients experience motor fluctuations, which are often accompanied by mood fluctuations. The nature of the relationship between motor and mood fluctuations remains unknown. It is suggested that the experience sampling method can reveal such associations on both a group and individual level. Revealing group patterns may enhance our understanding of symptom interactions and lead to more general treatment recommendations, whereas analyses in individual patients can be used to establish a personalized treatment plan.

**Objectives:**

To explore the usability of routinely collected experience sampling method data over a brief period of time to detect associations between motor fluctuations, affective state, and contextual factors in PD patients with motor fluctuations on a group level and on an individual level.

**Methods:**

Eleven patients with motor fluctuations collected data at 10 semirandom moments over the day for 5 consecutive days.

**Results:**

On a group level, multilevel analyses showed significant associations between all motor symptoms and positive affect. Being at home was associated with increased balance problems and rigidity. Analyses on an individual level revealed much less significant associations that mostly, but not always, were in line with the results on a group level.

**Conclusion:**

This exploratory study showed significant associations between affective state, motor symptoms, and contextual factors in a group of PD patients with motor fluctuations, but less so in individual patients. Given that the ultimate aim is to use the experience sampling method as an aid to personalize treatments, the sensitivity of the approach needs to be increased. © 2020 The Authors. *Movement Disorders* published by Wiley Periodicals, Inc. on behalf of International Parkinson and Movement Disorder Society.

The cornerstone of treatment for Parkinson's disease (PD) is levodopa replacement.^1^ Unfortunately, fluctuations in parkinsonian symptoms attributed to l‐dopa replacement may occur sometimes as early as a few months after initiation of l‐dopa treatment.^2^ These usually start with “end‐of‐dose wearing‐off,” but, eventually, as the disease progresses, lead to unpredictable fluctuations between *on* and *off* state in ∼50% of patients.^2^, ^3^ Presence of motor fluctuations is associated with lower quality of life and accounts for significant morbidity.^4^, ^5^, ^6^ Motor fluctuations are often accompanied by distressing mood fluctuations, such as fluctuations in anxiety or depressive symptoms.^7^, ^8^, ^9^ Because of the fluctuating nature of motor and mood symptoms, their frequency is difficult to capture in routine cross‐sectional assessments that are usually retrospective in nature.^10^, ^11^, ^12^ The experience sampling method (ESM) can overcome these limitations.^13^, ^14^ With ESM, experiences and behaviors, as well as moment‐to‐moment changes in physical and mental states and contextual factors in the subject's natural environment, can be sampled. Data are collected repeatedly and randomly during the day, for several days, thereby reducing recall bias.^11^, ^15^, ^16^ In a recent N = 1 study, we demonstrated that ESM data collected for 1 month is able to reveal longitudinal associations between motor symptoms and mood states and has the potential to be used as an aid to optimize individual treatment.^14^, ^17^ However, in routine patient care, much briefer sampling periods of 5 to 7 days are commonly used, given that longer periods of time are considered burdensome for patients.^14^ The question is whether brief periods of data collection generate enough power for the identification of significant associations between mood and motor symptoms. Given that the aim is to develop ESM as an aid to personalize treatment in individual patients, adequate power needs to be ensured.

The aim of the present study was to explore the usability of ESM data routinely collected over a brief period of time in PD patients suffering from motor fluctuations and to explore the sensitivity to detect associations between affective state, contextual factors, motor symptoms, and motor state on both a group and individual level. For that purpose, we explored: (1) associations between affective state, motor symptoms, and contextual factors in PD on a group level, as well as the role of motor (*on*/*off*) state in these associations, and (2) whether ESM data are sensitive enough to reveal similar associations in individual patients.

## Participants and Methods

### Design and Participants

This is a cross‐sectional, observational study. Twelve PD patients with motor fluctuations were recruited from the Movement Disorder clinic of Maastricht University Medical Center (MUMC+). Five of these were recruited in January and February 2015 and participated as part of a feasibility study of ESM in PD patients.^11^ Seven patients were additionally recruited between February 2017 and April 2017. Inclusion criteria were: a diagnosis of idiopathic PD according to the Queen Square Brain Bank diagnostic criteria^18^; the usage of a stable dose of l‐dopa or other antiparkinsonian medication for at least 1 month, the presence of motor fluctuations as indicated by part IV of the UPDRS, and an age between 50 and 80 years. Subjects with cognitive impairment, operationalized as a Mini‐Mental State Examination (MMSE) <26,^19^ were excluded in order to avoid problems with working with the mobile ESM application. Other exclusion criteria were: the presence of neurological disorders other than PD and excessive use or abuse of alcohol, drugs, or benzodiazepines. The institution's medical ethics committee ruled that collection of ESM data in the context of routine clinical outcome monitoring is exempt from ethical approval.

### Assessment

After informed consent was obtained, all patients underwent a baseline assessment, which involved the collection of demographic and disease‐related characteristics. All patients were tested in *on* state. The following instruments and scales were administered: the H & Y staging scale,^20^ the UPDRS part III^21^ to quantify motor symptoms, the MMSE^19^ to assess global cognitive function, the Parkinson Anxiety Scale (PAS)^22^ to assess anxiety symptoms, and the Beck Depression Inventory II (BDI‐II)^23^ to assess depressive symptoms. Quality of life was assessed using the Parkinson's Disease Questionnaire‐8 (PDQ‐8).^24^ The UPDRS‐III was administered by a neuropsychologist holding an International Parkinson and Movement Disorder Society (MDS)‐issued certificate of training in application of the UPDRS and ample experience in administering this instrument (A.M.). All other scales were administered by a trained medical student (R.v/dV.).

Using a mobile device with an ESM application (PsyMate; www.psymate.eu), participants were asked to answer several questions multiple times a day. During the baseline assessment, subjects received oral information and a demonstration to ensure that the ESM app was fully understood. Subjects were given the option to either install the ESM app on their own smartphone or to use an iPod‐touch with the PsyMate app provided by MUMC for the duration of the study. The following 5 consecutive days, the PsyMate generated auditory reminders (beeps) at unpredictable moments in each of ten 90‐minute time blocks between 7:30 am and 10:30 pm (10 beeps a day, during 5 days). After each reminder, subjects were asked to fill out a set of questions regarding motor, mood, and general symptoms, as well as contextual factors (see *ESM Questions*). Questions always appeared in the same order and were available for 15 minutes only in order to reduce recall bias. Answering the questionnaires takes ∼2 minutes. In addition, every morning and evening subjects were asked to fill out a morning and evening questionnaire, consisting of questions about the previous night and day, respectively.

Two days following the start of data collection, participants were contacted by telephone to inquire about any difficulties or concerns regarding the ESM application and questionnaires and to solve any problems, if necessary. Five days following the start of data collection, the data were uploaded in an anonymous central database and subjects returned the Ipod‐touch, if necessary.

### ESM Questions

The questions in the ESM protocol were based on a protocol used for routine outcome monitoring of the psychiatric outpatient department of our hospital. The protocol includes questions in several domains: context (eg, “Where am I?”); events (“the most important event since the last beep was”); mood (eg, “I feel happy”); and somatic (eg, “I am tired”). The protocol was supplemented with six PD‐specific items (motor symptoms): tremor; rigidity; walking problems; balance problems; dyskinesia (all scored on a 7‐point Likert scale, ranging from 1 = not at all to 7 = very much); and *on*/*off* state (*on* = 1, *off* = 0; Supporting Information Table S1).^11^ *On* and *off* states are defined subjectively by each individual subject. In general, in an *on* state the subject perceives his antiparkinson medication to be working properly in alleviating symptoms, while in an *off* state symptoms start to reappear.

Based on standard ESM mood items (also scored on a 7‐point Likert scale), two mood‐related variables reflecting positive and negative affect were computed. Positive affect was defined as the mean score of the items *cheerful*, *relaxed*, and *satisfied*. Negative affect was defined as the mean score of the items *insecure*, *irritated*, *lonely*, *anxious*, *down*, and *guilt*. Based on the questions “Where am I?” and “With who am I,” two contextual variables *at home* (at home = 1, not at home = 0) and *being alone* (alone = 1, not alone = 0) were computed.

### Statistical Analysis

All statistical analyses were performed using SPSS for Windows (version 24.0; SPSS, Inc., Chicago, IL)^25^ and Stata software (version 13; StataCorp LP, College Station, TX).^26^ Descriptive analyses were conducted to assess response rates. To assess usability on a group level, associations between affective state, contextual factors, motor symptoms, and *on*/*off* state were studied in the full data set. ESM data have a hierarchical structure because of within‐subject clustering: repeated measurements (level 1) are nested within individuals (level 2). Nesting of data violates the assumption of independent residuals, given that observations within subjects are more similar than observations among different subjects. Therefore, multilevel linear regression analyses were performed using the Stata mixed command with subject as the macro level. The following motor symptoms were the dependent variables in the regression models: tremor, rigidity, walking problems, balance problems, and dyskinesia. To control for time trends, a time variable was added to all regression models (beepcode=daynumber*10+beepnumber). Independent variables were positive and negative affect, as well as being at home and being alone.

First, in order to assess whether *on*/*off* state is a modifier in the association between affective state and contextual factors on the one hand and motor symptoms on the other hand, interaction effects of all independent variables with *on*/*off* state (*on* = 1, *off* = 0) were analyzed, including each independent variable in a separate model. Importantly, convergence was not achieved after including random slopes of the independent variable, *on*/*off* state and beepcode, and, consequently, only models including a random slope for beepcode were run. Given that this results in too optimistic *P* values, permutation testing was performed in case of a statistically significant result. Permutation testing obtains the distribution of regression coefficients under the null hypothesis. To obtain >1,000 valid *P* values, 1,100 permutation tests were performed. For this, the dependent variable was removed from the data and within‐subject randomly reshuffled and merged to the original data, while keeping the multilevel structure. Next, the regression coefficient obtained from the real multilevel regression analysis was placed on that normal distribution to obtain *P* values (ie, the proportion of times that the coefficient in the permuted data was as large as or larger than the observed coefficient; multiplied by 2 to obtain a two‐sided *P* value).^27^

Second, associations between the independent variables and the severity of motor symptoms independent of *on*/*off* state were analyzed in the full data set by multilevel linear regression analyses, including all independent variables in one model.

Third, in order to assess the usability of ESM on an individual level, linear regression analyses were performed in data from individual patients using the same variables included in the full data set. Moreover, graphs were generated to illustrate individual moment‐to‐moment fluctuations in mood and motor symptoms and *on*/*off* state.

## Results

### Participants and Descriptive Statistics

One participant was excluded from the analysis because of a response rate of 26%, which is below the generally accepted minimum response rate of 33% for ESM data sampling.^14^ Among the remaining 11 participants, a total of 412 questionnaires were completed, corresponding to a mean response rate of 74.9%. Between individual patients, the response rate varied from 42% to 94% (Supporting Information Table S2). Demographic and disease‐related characteristics of participants are summarized in Table 1. The study population consisted of 4 females and 7 males. Mean age was 62.3 years (standard deviation [SD] = 7.8); mean disease duration was 7 years (SD = 2.6), and mean UPDRS part III score was 24.7 (SD = 11.0). Median H & Y stage was 2 (range, 1.5–3.0).^20^ A total of 4 patients were on a stable dose of dopamine agonists during the course of the study (either ropinirole or pramipexole), 3 patients used l‐dopa/benserazide, 7 patients used l‐dopa/carbidopa, 1 patient used l‐dopa/carbidopa/entecapone, and 4 patients used amantadine. On the PAS, 5 subjects (participants 2, 3, 5, 7, and 10) scored above the cut‐off point of 14, indicating clinically relevant anxiety.^22^ On the BDI‐II, 4 subjects (participants 2, 3, 4, and 10) scored above the cut‐off point of 14 for mild depression.^23^ Mean scores for affective state and motor symptoms are shown in Table 2. Of the 412 completed questionnaires, 111 were completed in *off* state (27%) and 301 in *on* state (71%). Even though motor fluctuations were present, 1 patient did not report any *off* states. The percentage *on* varied from 46.7% to 100% in individual subjects.

**Table 1:** Demographic and disease‐related characteristics of participating patients

Sub.nr	Sex	Age (Years)	Disease dur. (Years)	Tremor Dom.	LEDD	UPDRS‐I	UPDRS‐II	UPDRS‐III	UPDRS‐IV	% OFF	H & Y	MMSE	PAS	BDI‐II	PDQ‐8
1	M	57	8	No	600	1	15	49	6	26 to 50	3	28	4	12	9
2	M	53	7	Yes	800	4	12	21	10	1 to 25	2.5	28	17	14	21
3	F	77	3	Yes	800	6	17	28	8	26 to 50	3	27	24	16	11
4	F	62	6	Yes	712.5	5	11	34	4	26 to 50	3	29	11	15	14
5	F	61	5	No	690	3	9	8	7	1 to 25	1.5	29	19	12	10
6	M	73	11	Yes	1,000	3	10	22	4	26 to 50	1.5	30	3	1	5
7	M	65	6	Yes	1,100	1	14	22	9	1 to 25	2,5	30	19	11	12
8	M	63	8	No	1,306	0	15	15	7	26 to 50	2	29	10	1	7
9	M	50	5	No	557	4	8	25	6	1 to 25	2	30	10	8	9
10	F	50	11	Yes	835	2	12	32	9	1 to 25	2	30	20	16	13
11	M	64	10	No	1,242	2	8	16	10	1 to 25	2	29	9	3	10

Sub.nr, Subject nr; Disease dur., disease duration; tremor dom., tremor‐dominant PD; LEDD, l‐dopa equivalent daily dose, % OFF; H&Y, Hoehn & Yahr staging system; PAS, Parkinson anxiety scale; BDI‐II, Beck Depression Inventory II; PDQ‐8, Parkinson's disease questionnaire‐8. UPDRS‐IV question 39.

**Table 2:** Descriptive statistics of ESM variables used in the analysis

	Mean	Median	SD	Variance	Range
Positive affect	4.55	5	1.40	1.97	1 to 7
Negative affect	3.33	1.17	0.55	0.31	1 to 5
Tremor	1.91	1	1.24	1.53	1 to 6
Rigidity	1.59	2	1.23	2.12	1 to 7
Balance problems	2.49	1	1.46	1.50	1 to 7
Walking problems	2.75	2	1.93	3.74	1 to 7
Dyskinesia	1.93	1	1.30	1.68	1 to 7

### Group‐Level Analyses

#### Effect Modification of *On*/*Off* State

Dyskinesia was not analyzed, given that 5 patients did not suffer from dyskinesia. Interaction analyses showed a statistically significant interaction between *on*/*off* state and being at home when analyzing tremor (χ^2^_(1)_ = 4.8; *P* = 0.028; Supporting Information Table S3). However, following permutation analysis, the association was no longer statistically significant (Supporting Information Table S4). There were no other significant interaction effects.

#### Associations Between Affective State, Contextual Factors, and Motor Symptom Severity

Multilevel linear regression analyses showed statistically significant negative associations between positive affect and all motor symptoms: Higher scores on positive affect were associated with lower self‐perceived severity of tremor, rigidity, balance problems, and walking problems. In addition, being at home was associated with increased balance problems and rigidity, whereas being alone was associated with reduced rigidity (Table 3).

**Table 3:** Associations between motor symptoms, mood states, and contextual factors (independent of *on*/*off* state)

	Tremor				Rigidity				Walking Problems				Balance Problems*			
	B	SE	*P* Value	95% CI	B	SE	*P* Value	95% CI	B	SE	*P* Value	95% CI	B	SE	*P* Value	95% CI
PA	–0.16	0.06	**0.007**	–0.28 to –0.05	–0.26	0.07	**0.000**	–0.39 to –0.12	–0.23	0.07	**0.001**	–0.16 to –0.01	–0.09	0.04	**0.032**	–0.16 to –0.01
NA	0.12	0.12	0.29	–0.11 to 0.35	0.28	0.14	***0.041*** *	0.01 to 0.55	0.18	0.13	0.17	–0.08 to 0.43	0.06	0.08	0.42	–0.08 to 0.21
At home	0.17	0.13	0.18	–0.08 to 0.43	0.41	0.15	**0.008**	0.10 to 0.71	0.23	0.14	0.11	–0.53 to 0.51	0.22	0.08	**0.010**	0.05 to 0.51
Being alone	–0.14	0.12	0.21	–0.37 to 0.09	–0.42	0.14	**0.002**	–0.69 to –0.02	–0.21	0.13	0.10	–0.46 to 0.04	–0.14	0.08	0.06	–0.29 to 0.01

Bold = significant result.

* This association was not significant anymore following sensitivity (permutation) analysis, *P* = 0.076.

PA, positive affect; NA, negative affect; CI, confidence interval.

### Individual‐Level Analyses

#### Associations Between Affective State, Contextual Factors, and Motor Symptom Severity

Individual moment‐to‐moment fluctuations in positive affect, negative affect, and motor symptoms are displayed in Supporting Information Figure S1. In 6 subjects (subjects 1, 6, 7, 8, 9, and 10), at least one statistically significant association was found. In general, these associations were in line with findings on a group level, with positive affect being associated with less motor symptoms. In 1 subject, positive affect was negatively associated with tremor, rigidity, and walking problems, whereas those same symptoms, as well as balance problems, were positively associated with negative affect (subject 10; Supporting Information Table S5).

Regarding contextual factors, analyses showed statistically significant reduced tremor (subject 8) and balance problems (subject 7) while being at home (Supporting Information Table S6). There was a statistically significant negative association in 2 other patients, with reduced tremor (subject 10) and rigidity (subject 9) while being alone. In 3 patients, only one regression coefficient is provided per outcome; this represents the association between being at home and the outcomes (see *Methodological Issues*).

## Discussion

The present study aimed to study whether routinely collected ESM data over a brief period of time can be used to study associations between affective state, contextual factors, motor symptoms, and motor state (*on*/*off*) in PD patients who suffer from motor fluctuations on a group and individual level. Revealing population based patterns may lead to more general treatment recommendations, whereas individual analyses can be used to establish a personalized treatment plan.^14^ Ideally, assessments are kept as brief as possible, in order to reduce the burden and improve compliance rates of patients.^14^

On the group level, ESM collected over a period of 5 consecutive days was able to identify clear associations between affective state, contextual factors, and motor symptoms. Higher scores on positive affect were associated with lower self‐perceived severity of tremor, rigidity, balance problems, and walking problems. Being at home was associated with more balance problems and rigidity in general. The observed associations were independent of whether a patient was in an *on* or *off* state. When analyzing data of individual patients, only a few subjects showed statistically significant associations between affective state, contextual factors, and motor symptoms, which were mostly, but not always, in the same direction as the findings on the group level.

#### Group Level

The finding that *on*/*off* state was not a modifier in the associations between affective state and motor symptoms was surprising. Given that mood symptoms are mostly related to *off* states,^9^ the relationship between positive or negative feelings and the severity of motor symptoms might be different when a patient is *off* compared to *on*, for example, there might be increased symptom focus in *off* state when experiencing negative emotions, or an increased symptom focus when *off* may lead to a stronger response in negative emotions. However, *off* states might have been under‐represented in the present study, leading to lack of power (see *Methodological Issues* below). Given that there are only a few reports examining motor fluctuations using ESM data in PD patients, it is hard to compare the present findings with results from other studies. A recent study by Fernie and colleagues examined emotional distress and motor states in PD patients using ESM data and found that episodic distress is greater during *off* states compared to *on* states, but worse during *off*‐coming‐*on* compared to *on*‐wearing‐*off* transitional states.^28^ Unfortunately, the present study could not identify these transitional states. Moreover, the study by Fernie and colleagues specifically focused on emotional distress, and their questionnaire did not include different motor symptoms or any items reflecting positive emotions.^28^

Interestingly, we observed that positive affect was associated with reduced self‐perceived symptom severity for all motor symptoms, whereas negative affect was not associated with any of the motor symptoms. Given that the present data are cross‐sectional, causal inferences cannot be made. Therefore, we do not know whether a better mood results in reduced self‐perceived severity of motor symptoms, or whether reduced self‐perceived severity of motor symptoms results in better mood. Nonetheless, it seems that the relationship between motor symptoms and positive affect is more significant in PD, compared to the relationship between negative affect and motor symptoms. This finding is in line with results from the network analysis we recently performed on ESM data from 1 PD patient, which aimed to identify longitudinal associations between motor symptoms and mood states.^17^ Whereas feeling “down” did not influence motor symptom severity, “cheerfulness” was significantly associated with better scores for tremor and rigidity in this N = 1 study.^17^ The associations between mood and motor states were independent of whether a patient subjectively perceived to be in an *on* or *off* state. The dosage and timing of medication may have served as a modifier between the observed associations. Previous studies have demonstrated mood changes related to l‐dopa infusion in a dose‐dependent fashion.^29^, ^30^, ^31^ For future research, it would be of clinical relevance to include questions regarding timing and dosage of medication. Moreover, most studies on the effects of affective state on motor symptoms in PD have focused on negative mood states, such as depression, anxiety, and apathy, ignoring positive affect.^9^ In the field of psychiatry, there is increasing evidence that the impact of interventions, both pharmacological and nonpharmacological, is mediated more by enhancing positive emotions than by reducing negative emotions.^32^, ^33^ It is therefore important to include positive affect in the design of future studies. Moreover, enhancing the frequency and persistence of positive affect in daily life might be a promising adjunctive nonpharmacological target for reducing the burden of motor fluctuations.

Analyses of the associations between contextual factors on the one hand and self‐perceived motor symptoms on the other hand showed that, overall, subjects perceived more rigidity and balance problems when at home. Possibly, patients have a tendency to stay at home when experiencing balance problems. Balance problems are among the most distressing symptoms related to PD,^34^ given that they are often accompanied by fear of falling,^35^ thereby limiting a patient's mobility and independence. Alternatively, being at home could also contribute to increased symptom focus or an increased chance of being confronted with balance problems attributed to the presence of more obstacles in smaller rooms. The negative association between rigidity and being alone is more difficult to explain, given that there were no other statistically significant associations between being alone and motor symptoms. In general, context‐specific associations based on analyses of a group of patients are difficult to interpret without patient feedback. Context is an important consideration for many symptoms, and the purpose of including contextual factors in ESM sampling strategies is to reveal complex environmental dynamics that can potentially help individuals to optimize environmental interactions and benefit coping strategies.^32^

#### Individual Level

Overall, we found less clear associations when analyzing routinely collected ESM data in individual patients. Although most associations between being at home and balance problems in individual patients were also positive but not significant, to our surprise, the only patient with a statistically significant association was in opposite direction compared to the findings on a group level. Regarding positive and negative affect, most associations were in the same direction compared to the group analyses, but not all. One could assume that those subjects that suffered from clinically relevant anxiety and/or depression at baseline would show more significant associations between negative affect and motor symptoms. Except for subject 10, who showed statistically significant associations for both positive and negative affect, there was only 1 other patient with baseline anxiety in whom negative affect was associated with increased balance problems. We think the most likely explanation for the failure to identify significant associations between symptoms in individual patients is lack of power.

On the other hand, the graphs were able to display moment‐to‐moment fluctuations in mood and motor symptoms. For clinical practice, this might provide the clinician sufficient information that was otherwise not available through retrospective interviews or routine cross‐sectional assessments in which a single assessment of symptom severity is considered representative for a longer time frame. Graphical representations of symptoms might also give the patient more insight into particular circumstances in which fluctuations occur or are perceived as more disabling, especially when fluctuations have become unpredictable. Apart from using ESM data to visualize and monitor symptom change following medication adjustments, it can also be used to guide collaborate decision making between the patient and clinician, for example determining which information is most important to monitor by ESM or to support treatment target selection.^14^, ^32^

#### Methodological Issues

This study has several limitations. First, the sample size was small. Given that ESM generates a large number of data because of intensive data collection, we were able to reveal group‐level associations. However, for the individual analyses, 5 days of ESM data collection is probably too short to obtain sufficient power. In order to be applicable in routine patient care, the sensitivity of the method needs to be increased. This may be done by collecting data over a longer period of time, as previously shown in our N = 1 study, by increasing the compliance or by making the answers more sensitive to change (eg, by increasing the number of potential scores on the Likert item or by using visual analogue scale scores). Similarly, the finding that *on*/*off* state was not a modifier in the studied associations might also be attributable to lack of power, given that only 27% of completed questionnaires were in the *off* state. Whereas highly fluctuating states might be undersampled with an ESM sampling frequency of 10 times per day, slowly fluctuating states might be oversampled.^14^ It is possible that with the current ESM protocol motor fluctuations were not adequately represented. For that reason, we did not include *on*/*off* state in the analyses of individual subjects. In order to adequately sample *on*/*off* fluctuations, the interval between assessments should be adapted to fit the frequency and duration of the *off* periods. An alternative approach would be to allow the patient to complete “patient‐generated beep assessments” during *off* periods or to program the assessments at fixed times. However, with the latter method, one may run the risk of missing the fluctuating nature of symptoms. Third, we did not include intermediate or transitional motor states, such as *on*‐wearing‐*off* or *off*‐coming‐*on*, which might reveal different and potential relevant associations between affective states and motor symptoms. In addition, *on*/*off* state was defined subjectively by each individual subject. The use of wearable devices to objectify motor fluctuations may be a valuable addition in this respect. Recently, a study showed that combining wearable sensors with ESM data is feasible.^36^ Fourth, the current study had a cross‐sectional approach, which makes it impossible to study assess temporal or causal associations. Studying temporal associations requires much longer periods of data collection, which may be too long to be implemented in routine patient care. In the context of an N = 1 study, we collected ESM data for a longer period of time—1 month—in a PD patient. Although we were able to reveal longitudinal associations between affective state and motor symptoms in this patient, his response rate declined >50% over the course of 30 days, which may reflect the burden that patients experience when collecting data over a longer period of time.^17^ Fifth, four patients used a (stable) dose of dopamine agonists during the study, which may be a confounder when measuring mood states. With the analysis performed in the present study, it is not possible to correct for such confounders. For future research and for clinical practice, it would be useful to record the time of medication intake, as well as missed or delayed intakes, to monitor the effect of dopaminergic treatment on motor symptoms or mood fluctuations. Sixth, we could not include random effects for the slopes of all predictor variables included in the models of the multilevel linear regression analysis; that is, both the interaction analyses and the main effects, which might lead to an overestimation of *P* values. Moreover, the assumption of normality of residuals was not met for all variables (eg, for balance problems). To obtain valid and interpretable results, we performed permutation testing. Given that these sensitivity analyses revealed similar results in all but 2 cases (the interaction between *on*/*off* state and being at home when analyzing tremor and the association between rigidity and negative affect), we adopted the results from the multilevel linear regression analyses and did not perform further permutation testing on data of individual subjects. Seventh, in 3 subjects, the contextual factor *being alone* was removed from the linear regression model because those subjects were always alone when at home and in company when not at home. Thus, the regression coefficients presented in Supporting Information Table S6 were, in fact, the association between being home alone and the respective outcomes. Finally, the results are based on a priori selected variables that aimed to represent positive and negative mood states, as well as contextual factors, and should therefore be considered with caution. Selecting different (combinations of) items might lead to different results. For clinical practice, items are ideally selected based on the specific situation of the patient in a collaborative process between clinician and patient.

## Conclusion

The ultimate goal is to use ESM in a nonburdening way in routine patient care as a tool to facilitate patient self‐monitoring and as a guidance for clinicians to personalize treatment. Whereas in this study ESM collected over a period of 5 days appeared sensitive enough to reveal associations between motor symptoms, mood states, and contextual factors on a group level, this approach was not powerful enough to reveal an adequate number of relevant associations on an individual level. This implies that the method must be made more sensitive for application in routine clinical practice. Given that ESM will probably play a more important role in future studies as well as in clinical practice, we argue that there is an urgent need of harmonization, validation, and optimization of this technique for use in PD patients.

## Author Roles

(1) Research Project: A. Conception, B. Organization, C. Execution; (2) Statistical Analysis: A. Design, B. Execution, C. Review and Critique; (3) Manuscript Preparation: A. Writing of the First Draft, B. Review and Critique.

A.E.P. Mulders: 1C, 2A, 2B, 2C, 3A

R.M.J. van der Velden: 1A, 1B, 1C, 3B

M. Drukker: 2A, 2B, 2C, 3B

M.P.G. Broen: 1C, 3B

M.L. Kuijf: 1B, 3C

A.F.G. Leentjens: 1A, 2A, 1B, 3B

## Financial Disclosures

A.F.G.L. has received a research grant from the Michael J Fox Foundation as well as royalties from Springer media. He received payment from Elsevier Inc. as Editor‐in‐Chief of the *Journal of Psychosomatic Research* up until December 2017.

## Supporting information


**Supplementary table 1:** ESM questionnaire
**Supplementary table 2:** Overview of response rates
**Supplementary table 3.** Interaction effects ON/OFF state
**Supplementary table 4.** P‐values original analyses and permutation analyses
**Supplementary table 5:** Associations between motor symptoms and mood states in individual patients
**Supplementary table 6:** Associations between motor symptoms and contextual factors in individual patients
**Supplementary figure 1:** Moment‐to‐moment fluctuations in positive and negative affect and in tremor, rigidity, balance problems, walking problems and dyskinesia in individual patients
