# Supplementary material for: Usability of the Experience Sampling Method in Parkinson's Disease on a Group and Individual Level
Source: Mov Disord. 2020 May 30;35(7):1145–52. doi: 10.1002/mds.28028 (PMC7496752; doi:10.1002/mds.28028)

**Supplementary table 1:** ESM questionnaire

| **Domain** | **Item** | **Description** | **Score** |
| --- | --- | --- | --- |
| **Mood** | 1 | I feel happy | 1-7 (1= not, 4= moderate, 7= very) |
|  | 2 | I feel insecure | 1-7 |
|  | 3 | I feel relaxed | 1-7 |
|  | 4 | I feel irritated | 1-7 |
|  | 5 | I feel satisfied | 1-7 |
|  | 6 | I feel lonely | 1-7 |
|  | 7 | I feel afraid | 1-7 |
|  | 8 | I feel down | 1-7 |
|  | 9 | I feel guilty | 1-7 |
|  | 10 | I rack my brain | 1-7 |
|  | 11 | I feel suspicious | 1-7 |
|  | 12 | I feel threatened | 1-7 |
|  | 13 | In general, I feel well | 1-7 |
| **Parkinson** | 14 | Currently I am: | 0=”OFF”, 1=”ON” |
|  | 15 | I experience tremor | 1-7 |
|  | 16 | I experience rigidity | 1-7 |
|  | 17 | Walking is difficult | 1-7 |
|  | 18 | I experience balance problems | 1-7 |
|  | 19 | I experience dyskinesia | 1-7 |
|  | 20 | Personal complaints: | Open question |
| **Context** | 21 | Currently I am: | Resting/working/housework/hygiene/  eating,drinking/relaxing/conversating/other |
|  | 22 | I rather do something else | 1-7 |
|  | 23 | Where am I? | Home/work/friends place/public place/on the go |
|  | 24 | With who am I? | Nobody/partner/family/friends/collegues/strangers |
|  | 25 | I find this pleasant | 1-7 |
| **Somatic** | 26 | I am hungry | 1-7 |
|  | 27 | Since the last beep I used: | Nothing/caffeine/nicotine/alcohol/medication/cannabis/  food/other |
|  | 28 | I am tired | 1-7 |
|  | 29 | I am not feeling well | 1-7 |
|  | 30 | I have pain | 1-7 |
|  | 31 | I experience: | Headache/abdominal pain/breathing difficulties/muscle ache/obstipation/tinnitus/dizziness/ dyspnea/ palpitations/incontinence |
| **Events** | 32 | The most important event since the last beep was: | Open question |
|  | 33 | The event was: | -3 - 3 (-3 = very displeasing, 0 = neutral, 3 = very pleasant ) |
|  | 34 | The event was: | -3 - 3 (-3 = not important, 0 = neutral, 3 = very important) |
|  | 35 | The event was under my control | 1-7 |
|  | 36 | I expected this event | 1-7 |
|  | 37 | This beep was unpleasant | 1-7 |

**Supplementary table 2:** Overview of response rates

| **Subject** | **Completed questionnaires (nr)** | **Response percentage (%)** | **ON / OFF (nr)** | **Percentage ON (%)** |
| --- | --- | --- | --- | --- |
| 1 | 21 | 42 | 19 / 2 | 90.5 |
| 2 | 26 | 52 | 23 / 3 | 88.5 |
| 3 | 42 | 84 | 42 / 0 | 100 |
| 4 | 35 | 70 | 18 / 17 | 51.4 |
| 5 | 46 | 92 | 28 / 18 | 60.9 |
| 6 | 47 | 94 | 27 / 20 | 57.4 |
| 7 | 30 | 60 | 14 / 16 | 46.7 |
| 8 | 39 | 78 | 26 / 13 | 66.7 |
| 9 | 46 | 92 | 42 / 4 | 91.3 |
| 10 | 45 | 90 | 36 / 9 | 80 |
| 11 | 35 | 70 | 26 / 9 | 74.3 |
| Total | 412 | 74.9 | 301 / 111 | 73.1 |

**Supplementary table 3.** Interaction effects ON/OFF state

|  | **Tremor** | | | | **Rigidity** | | | | **Walking problems** | | | | **Balance problems** | | | |
| --- | --- | --- | --- | --- | --- | --- | --- | --- | --- | --- | --- | --- | --- | --- | --- | --- |
|  | **B** | **SE** | **p** | **95% CI** | **B** | **SE** | **p** | **95% CI** | **B** | **SE** | **p** | **95% CI** | **B** | **SE** | **p** | **95% CI** |
| **PA** | 0.04 | 0.07 | 0.59 | -0.11 - 0.18 | -0.08 | 0.09 | 0.39 | -0.10 - 0.02 | -0.15 | 0.09 | 0.07 | -0.32 - 0.02 | -0.05 | 0.05 | 0.32 | -0.16 - 0.01 |
| **NA** | 0.08 | 0.19 | 0.69 | -0.29 - 0.45 | -0.26 | 0.23 | 0.25 | -0.72 - 0.19 | -0.09 | 0.22 | 0.70 | -0.52 - 0.35 | 0.16 | 0.14 | 0.23 | -0.10 - 0.43 |
| **At home** | -0.54 | 0.25 | **0.028** | -.102 - .057 | -0.35 | 0.30 | 0.25 | -0.25 - 0.94 | 0.31 | 0.29 | 0.29 | -0.26 - 0.05 | 0.25 | 0.18 | 0.15 | -0.09 - 0.60 |
| **Being alone** | -0.24 | 0.20 | 0.24 | -0.63 - 0.16 | 0.33 | 0.25 | 0.19 | -0.16 - 0.81 | -0.04 | 0.24 | 0.87 | -0.50 - 0.42 | 0.10 | 0.15 | 0.27 | -0.12 - 0.43 |
| PA = positive affect, NA = negative affect, Bold = significant result p > 0.05 | | | | | | | | | | | | | | | | |

**Supplementary table 4.** P-values original analyses and permutation analyses

|  | **Tremor – ON/OFF** | | **Tremor** | | **Rigidity** | | **Walking problems** | | **Balance problems** | |
| --- | --- | --- | --- | --- | --- | --- | --- | --- | --- | --- |
|  | **P** | **P-PT** | **P** | **P- PT** | **P** | **P- PT** | **P** | **P- PT** | **P** | **P- PT** |
| **PA** | 0.59 | 0.99 | 0.007 | 0.002 | <0.001 | 0.006 | 0.001 | <0.001 | 0.032 | 0.039 |
| **NA** | 0.69 | 0.54 | 0.29 | 0.21 | 0.041 | 0.076 | 0.17 | 0.19 | 0.42 | 0.37 |
| **At home** | 0.028 | 0.99 | 0.13 | 0.14 | 0.008 | 0.001 | 0.11 | 0.11 | 0.010 | 0.013 |
| **Being alone** | 0.24 | 0.99 | 0.12 | 0.23 | 0.002 | <0.001 | 0.10 | 0.08 | 0.06 | 0.06 |
| PA = positive affect, NA = negative affect, PT = permutation testing | | | | | | | | | | |

**Supplementary table 5:** Associations between motor symptoms and mood states in individual patients

|  | **Symptom** | **Mood** | | | | | | | | | |
| --- | --- | --- | --- | --- | --- | --- | --- | --- | --- | --- | --- |
| **Pt**  **+ N** |  | **Positive affect** | | | |  | **Negative affect** | | | | |
|  |  | **B** | **SE** | **p** | **95% CI** |  | **B** | **SE** | **p** | **95% CI** | |
| **1**  **N=21** | Tremor | .102 | .403 | 0.804 | -.753-.957 |  | -.224 | .733 | 0.764 | -1.778-1.330 | |
|  | Rigidity | -.473 | .505 | 0.363 | -1.544-.598 |  | 1.862 | .918 | 0.060 | -.084-3.808 | |
|  | Walking problems | -1.027 | .402 | **0.021** | -1.880--.174 |  | 1.070 | .731 | 0.163 | -.481–2.620 | |
|  | Balance problems | -.431 | .322 | 0.199 | -1.113-.251 |  | .688 | .585 | 0.257 | -.552–1.927 | |
| **2** ^ab^  **N=26** | Tremor | -.138 | .448 | 0.762 | -1.073-.797 |  | -.425 | .617 | 0.498 | | -1.771-.861 |
|  | Rigidity | .078 | .414 | 0.853 | -.786-.942 |  | -.196 | .570 | 0.735 | | -1.384-.993 |
|  | Walking problems | -.181 | .162 | 0.277 | -.518-.156 |  | -.116 | .222 | 0.607 | | -.580-.347 |
|  | Balance problems | -.095 | .229 | 0.681 | -.574-.383 |  | .0251 | .315 | 0.937 | | -.632-.683 |
| **3** ^ab^  **N=42** | Tremor | .025 | .092 | 0.079 | -.161-.210 |  | .152 | .203 | 0.457 | | -.258-.563 |
|  | Rigidity | .042 | .204 | 0.838 | -.372-.456 |  | .248 | .452 | 0.587 | | -.668-1.163 |
|  | Walking problems | .039 | .175 | 0.825 | -.315-.393 |  | .321 | .386 | 0.412 | | -.462–1.104 |
|  | Balance problems | .073 | .086 | 0.401 | -.101-.246 |  | -.126 | .189 | 0.509 | | -.509-.257 |
| **4** ^b^  **N=35** | Tremor | .568 | .315 | 0.083 | -.078–1.213 |  | .768 | .838 | 0.367 | | -.947–2.483 |
|  | Rigidity | -.291 | .310 | 0.356 | -.925-.344 |  | -.014 | .824 | 0.986 | | -1.699–1.670 |
|  | Walking problems | .079 | .337 | 0.815 | -.609-.768 |  | .393 | .895 | 0.664 | | -1.436–2.223 |
|  | Balance problems | .025 | .050 | 0.625 | -.078-.127 |  | -.035 | .133 | 0.794 | | -.307-.237 |
| **5** ^a^  **N=46** | Tremor | -.085 | .042 | 0.050 | -.169--.001 |  | .056 | .052 | 0.284 | | -.049-.162 |
|  | Rigidity | -.372 | .202 | 0.072 | -.780-.035 |  | -.263 | .251 | 0.300 | | -.771-.244 |
|  | Walking problems | -.252 | .178 | 0.165 | -.612-.108 |  | -.29 | .222 | 0.200 | | -.737-.159 |
|  | Balance problems^c^ | . | . | . | . |  | . | . | . | | . |
| **6**  **N=47** | Tremor | .105 | .409 | 0.798 | -.721-.931 |  | -2.06 | 1.43 | 0.159 | | -4.95-.837 |
|  | Rigidity | -.111 | .198 | 0.577 | -.511-.286 |  | -1.483 | .692 | **0.038** | | -2.882--.084 |
|  | Walking problems | -.148 | .301 | 0.627 | -.755-.460 |  | .725 | 1.054 | 0.495 | | -1.403–2.855 |
|  | Balance problems | .076 | .104 | 0.468 | -.133-.286 |  | -.244 | .364 | 0.506 | | -.979-.490 |
| **7** ^a^  **N=30** | Tremor | -2.42 | .756 | **0.004** | -3.977--.862 |  | .083 | 1.38 | 0.952 | -2.760–2.927 | |
|  | Rigidity | -.969 | .466 | **0.048** | -1.928 -.010 |  | 1.72 | .850 | 0.054 | -.030–3.472 | |
|  | Walking problems | -1.032 | .591 | 0.093 | -2.250-.187 |  | -.093 | .108 | 0.932 | -2.317–2.131 | |
|  | Balance problems | -.070 | .217 | 0.750 | -.516-.377 |  | 1.190 | .396 | **0.006** | .375–2.005 | |
| **8**  **N=39** | Tremor | -.001 | .053 | 0.980 | -.109-.106 |  | .109 | .613 | 0.860 | -1.137–1.356 | |
|  | Rigidity | -.679 | .425 | 0.120 | -1.543-.186 |  | -2.911 | 4.918 | 0.558 | -12.91–1.094 | |
|  | Walking problems | -.702 | .427 | 0.110 | -1.571–.167 |  | -1.926 | 1.945 | 0.699 | -11.986–8.134 | |
|  | Balance problems | -.636 | .310 | **0.049** | -1.268--.004 |  | 1.977 | 3.598 | 0.586 | -5.343–9.296 | |
| **9**  **N=46** | Tremor | -.085 | .054 | 0.125 | -.194-.025 |  | -.010 | .085 | 0.908 | -.182-.162 | |
|  | Rigidity | -.694 | .206 | **0.002** | -1.110--.278 |  | .069 | .324 | 0.833 | -.588-.725 | |
|  | Walking problems | -.417 | .143 | **0.006** | -.705-.128 |  | .093 | .225 | 0.683 | -.363-.548 | |
|  | Balance problems | -.241 | .099 | **0.020** | -.441--.040 |  | -.016 | .157 | 0.919 | -.332-.300 | |
| **10** ^ab^  **N=45** | Tremor | -.104 | .246 | **0.000** | -1.536--.541 |  | 1.91 | .634 | **0.004** | .632-3.195 | |
|  | Rigidity | -.369 | .131 | **0.008** | -.631--.103 |  | .751 | .336 | **0.031** | .070–1.431 | |
|  | Walking problems | -.235 | .100 | **0.025** | -.438--.032 |  | .612 | .259 | **0.023** | .089–1.126 | |
|  | Balance problems | .058 | .091 | 0.529 | -.126-.242 |  | .624 | .234 | **0.011** | .150–1.098 | |
| **11**  **N=35** | Tremor | -.237 | .179 | 0.196 | -.602-.129 |  | .256 | .337 | 0.452 | -.432-.945 | |
|  | Rigidity | -.236 | .313 | 0.457 | -.876-.404 |  | .959 | .590 | 0.115 | -.247-.2.163 | |
|  | Walking problems | -.157 | .304 | 0.610 | -.778-.465 |  | 1.101 | .572 | 0.064 | -.070-.2.271 | |
|  | Balance problems | .033 | .149 | 0.826 | .-.272-.339 |  | .476 | .281 | 0.101 | -.099-.1.052 | |

N = number of completed questionnaires. Each participant received 50 assessments over the course of 5 days. Bold= significant result p > 0.05

^a^ Subject suffered from clinically relevant anxiety as objectified by the Parkinson Anxiety Scale^20^

^b^ Subject suffered from clinically relevant depression as objectified by the Beck Depression Inventory-II^21^

^c^ Subject did not experience any balance problems during ESM data collection (score =1)

**Supplementary table 6:** Associations between motor symptoms and contextual factors in individual patients

|  | **Symptom** | **Contextual factors** | | | | | | | | |
| --- | --- | --- | --- | --- | --- | --- | --- | --- | --- | --- |
| **Pt**  **+ N** |  | **At home** | | | |  | **Being alone** | | | |
|  |  | **B** | **SE** | **p** | **95% CI** |  | **B** | **SE** | **p** | **95% CI** |
| **1**  **N=21** | Tremor | .617 | .596 | 0.316 | -.647–1.881 |  | . | . | . | . |
|  | Rigidity | .392 | .746 | 0.607 | -1.191–1.974 |  | . | . | . | . |
|  | Walking problems | .868 | .595 | 0.164 | -.393–2.129 |  | . | . | . | . |
|  | Balance problems | .685 | .476 | 0.169 | -.323–1.693 |  | . | . | . | . |
| **2** ^ab^  **N=26** | Tremor | .046 | .656 | 0.945 | -1.322–1.414 |  | .449 | .696 | 0.527 | -1.004–1.901 |
|  | Rigidity | 1.155 | .606 | 0.071 | -.10–2.419 |  | .242 | .643 | 0.711 | -1.100–1.584 |
|  | Walking problems | .017 | .236 | 0.944 | -.476-.509 |  | -.133 | .251 | 0.602 | -.656-.390 |
|  | Balance problems | .680 | .335 | 0.056 | -.020–1.379 |  | .054 | .356 | 0.882 | -.689-.796 |
| **3** ^ab^  **N=42** | Tremor | .680 | .395 | 0.093 | -.119-1.480 |  | . | . | . | . |
|  | Rigidity | .093 | .880 | 0.916 | -1.689–1.876 |  | . | . | . | . |
|  | Walking problems | -.017 | .752 | 0.982 | -1.541–1.508 |  | . | . | . | . |
|  | Balance problems | .116 | .368 | 0.755 | -.631-.862 |  | . | . | . | . |
| **4** ^b^  **N=35** | Tremor | 1.114 | .921 | 0.236 | -.769–2.998 |  | -.780 | .402 | 0.062 | -1.603-.043 |
|  | Rigidity | .462 | .905 | 0.613 | -1.388–2.313 |  | -.607 | .395 | 0.135 | -1.415-.201 |
|  | Walking problems | -.405 | .983 | 0.683 | -2.415–1.604 |  | .423 | .429 | 0.333 | -.522–1.301 |
|  | Balance problems | .094 | .146 | 0.524 | -.204-.393 |  | -.063 | .064 | 0.332 | -.193-.068 |
| **5** ^a^  **N=46** | Tremor | .109 | .074 | 0.148 | -.040-.259 |  | -.105 | .079 | 0.189 | -.264-.054 |
|  | Rigidity | -.172 | .357 | 0.633 | -.893-.549 |  | -.066 | .380 | 0.863 | -.834-.702 |
|  | Walking problems | -.206 | .315 | 0.516 | -.844-.431 |  | -.104 | .336 | 0.759 | -.783-.575 |
|  | Balance problems | . | . | . | . |  | . | . | . | . |
| **6**  **N=47** | Tremor | -.465 | .433 | 0.289 | -1.340-.410 |  | -.203 | .316 | 0.525 | -.842-.436 |
|  | Rigidity | -.406 | .209 | 0.059 | -.829-.017 |  | -.218 | .153 | 0.162 | -.527-.091 |
|  | Walking problems | -.052 | .319 | 0.870 | -.696-.591 |  | .022 | .233 | 0.924 | -.448-.492 |
|  | Balance problems | .072 | .110 | 0.516 | -.150-.294 |  | .012 | .080 | 0.878 | -.150-.175 |
| **7** ^a^  **N=30** | Tremor | .375 | 1.030 | 0.719 | -1.746–2.497 |  | . | . | . | . |
|  | Rigidity | .290 | .634 | 0.651 | -1.016–1.598 |  | . | . | . | . |
|  | Walking problems | .923 | .805 | 0.263 | -.736-.583 |  | . | . | . | . |
|  | Balance problems | -.700 | .295 | **0.026** | -1.309--.092 |  | . | . | . | . |
| **8**  **N=39** | Tremor | -.242 | .084 | **0.007** | -.414--.070 |  | .061 | .079 | 0.446 | -.100-.223 |
|  | Rigidity | 1.183 | .680 | 0.091 | -.201 2.566 |  | -.734 | .637 | 0.257 | -2.030-.561 |
|  | Walking problems | 1.186 | .684 | 0.092 | -.206–2.577 |  | -.566 | .640 | 0.383 | -1.869-.736 |
|  | Balance problems | .948 | .497 | 0.066 | -.064–1.96 |  | -.457 | .465 | 0.334 | -1.405-.491 |
| **9**  **N=46** | Tremor | .251 | .199 | 0.215 | -.152-.654 |  | -.125 | .182 | 0.496 | -.492-.242 |
|  | Rigidity | 1.518 | .758 | 0.052 | -.015–3.050 |  | -1.514 | .691 | **0.034** | -2.910--.118 |
|  | Walking problems | .758 | .52 | 0.157 | -.306–1.822 |  | -.719 | .479 | 0.141 | -1.688-.250 |
|  | Balance problems | .369 | .366 | 0.319 | -.370–1.109 |  | -.362 | .333 | 0.284 | -1.035-.312 |
| **10** ^ab^  **N=45** | Tremor | .593 | .414 | 0.161 | -.246–1.430 |  | -.751 | .293 | **0.014** | -1.342--.158 |
|  | Rigidity | .290 | .220 | 0.195 | -.155-.735 |  | -.068 | .155 | 0.666 | -.382-.247 |
|  | Walking problems | .096 | .169 | 0.572 | -.246-.439 |  | .035 | .120 | 0.768 | -.206-.277 |
|  | Balance problems | -.085 | .153 | 0.583 | -.395-.225 |  | .008 | .108 | 0.939 | -.211-.227 |
| **11**  **N=35** | Tremor | .276 | .527 | 0.605 | -.802–1.353 |  | .195 | .347 | 0.578 | -.515-.906 |
|  | Rigidity | .909 | .922 | 0.322 | -.977–2.796 |  | -.669 | .608 | 0.280 | -1.913-.575 |
|  | Walking problems | .652 | .896 | 0.472 | -1.180–2.484 |  | -.709 | .591 | 0.239 | -1.197-.499 |
|  | Balance problems | .132 | .440 | 0.767 | -.769–1.032 |  | .419 | .290 | 0.160 | -.175–1.013 |
| N = number of completed questionnaires. Each participant received 50 assessments over the course of 5 days. Bold = significant result p > 0.05. In 3 subjects (1, 3 and 7) ‘being alone’ was removed from the model because of a 100% correlation with ‘being at home’  ^a^ Subject suffered from clinically relevant anxiety as objectified by the Parkinson Anxiety Scale^20^  ^b^ Subject suffered from clinically relevant depression as objectified by the Beck Depression Inventory-II^21^  ^c^ Subject did not experience any balance problems during ESM data collection (score =1) | | | | | | | | | | |

**Supplementary figure 1:** Moment-to-moment fluctuations in positive and negative affect and in tremor, rigidity, balance problems, walking problems and dyskinesia in individual patients


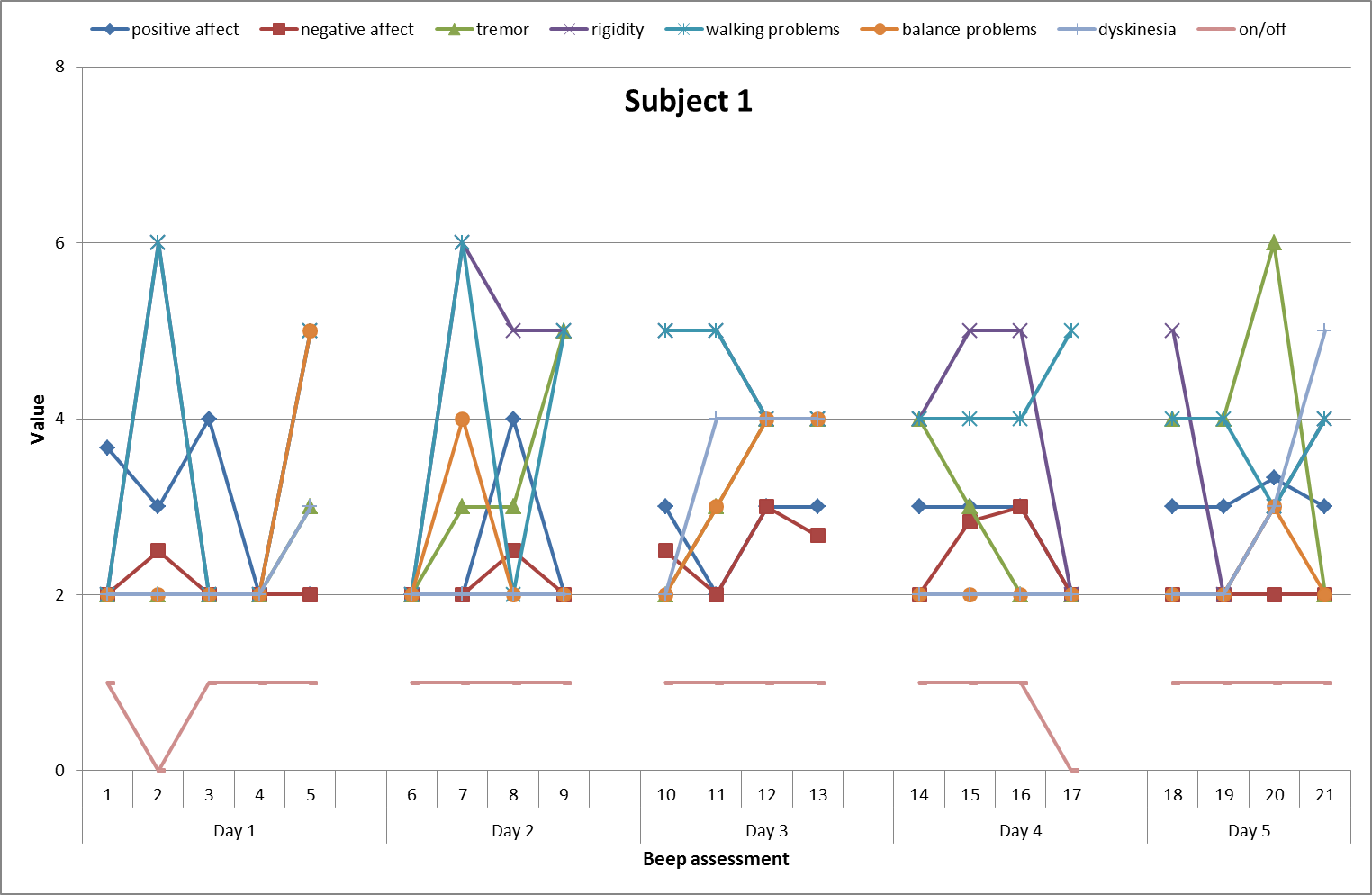


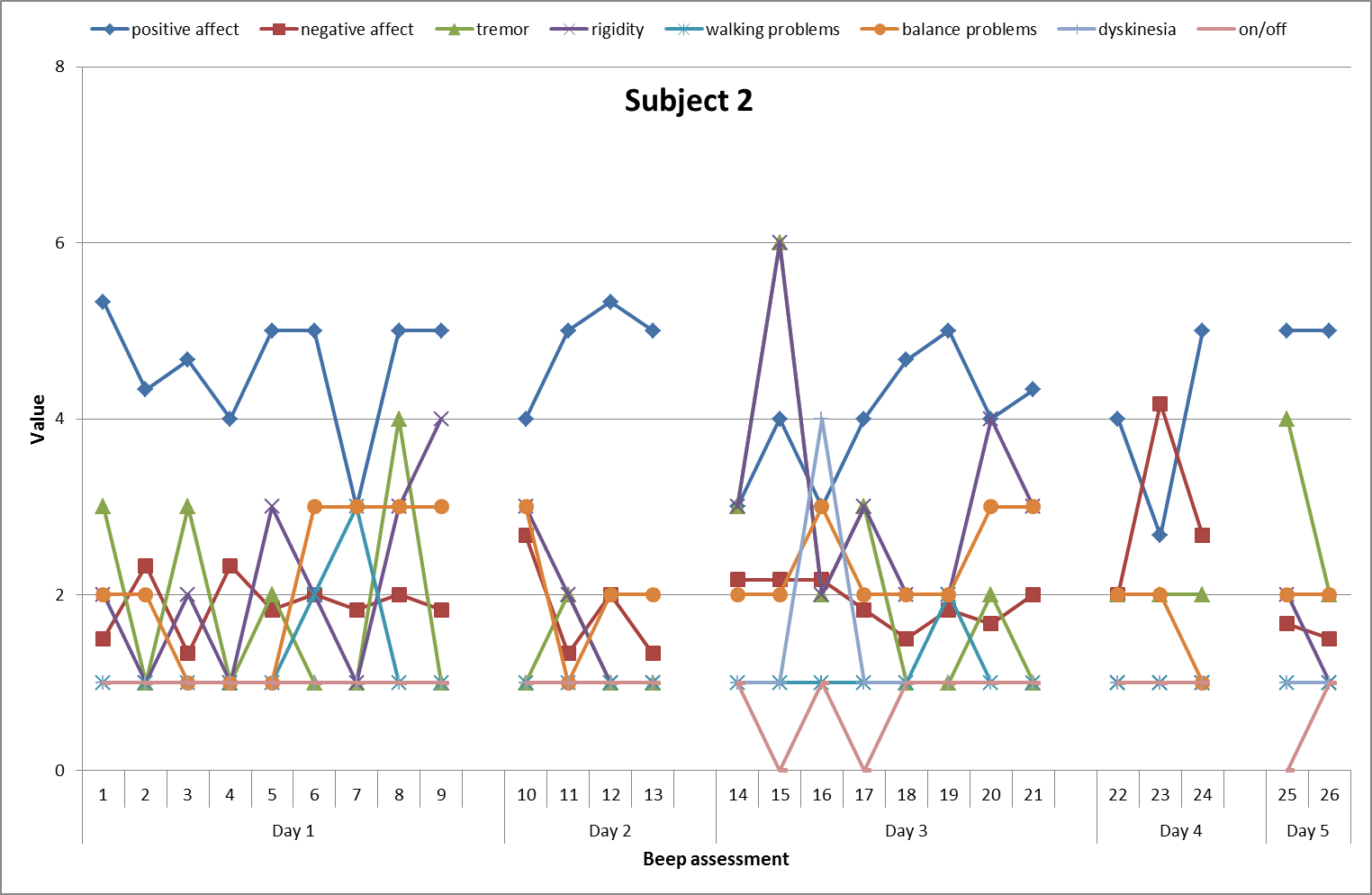


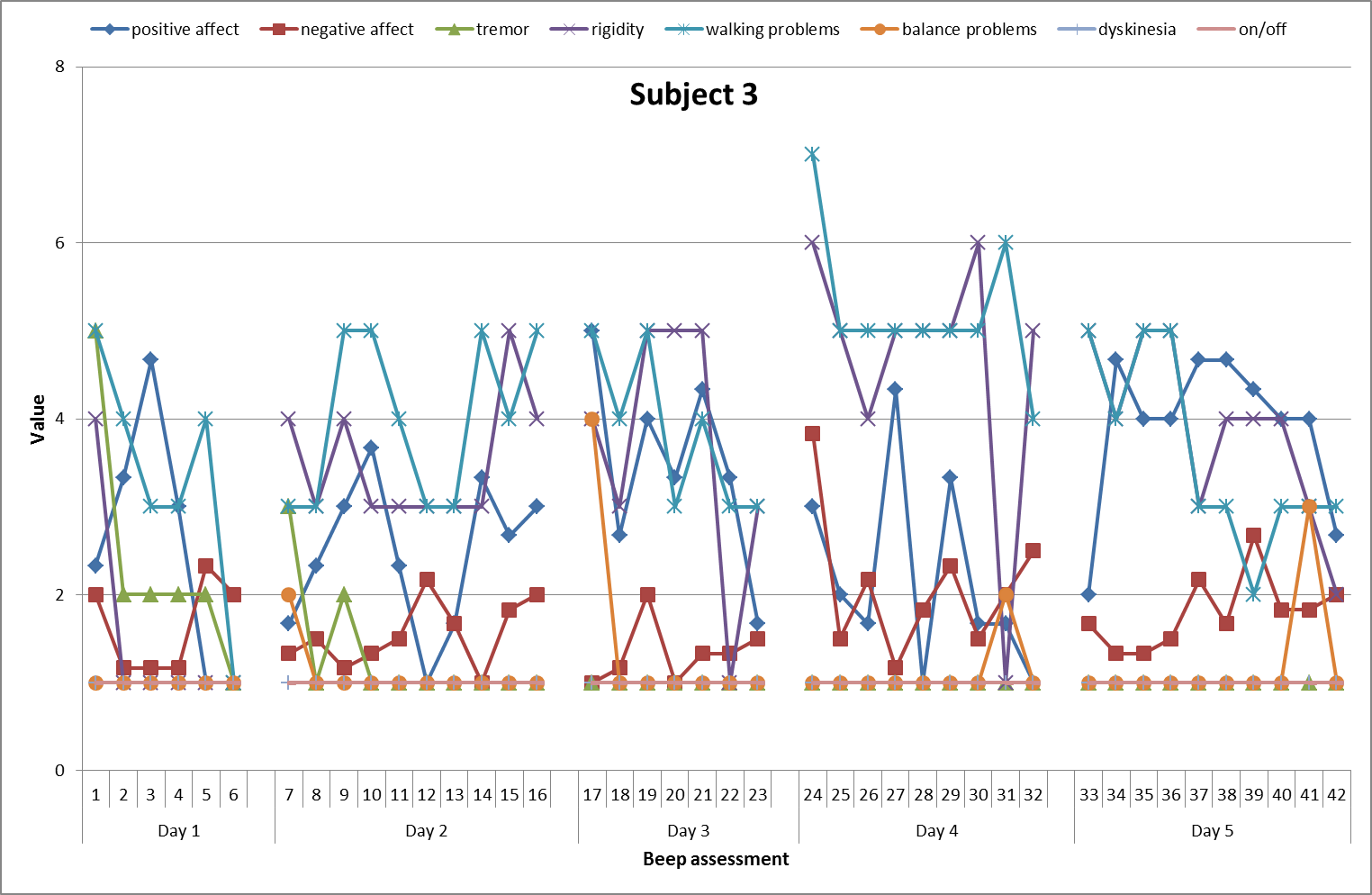


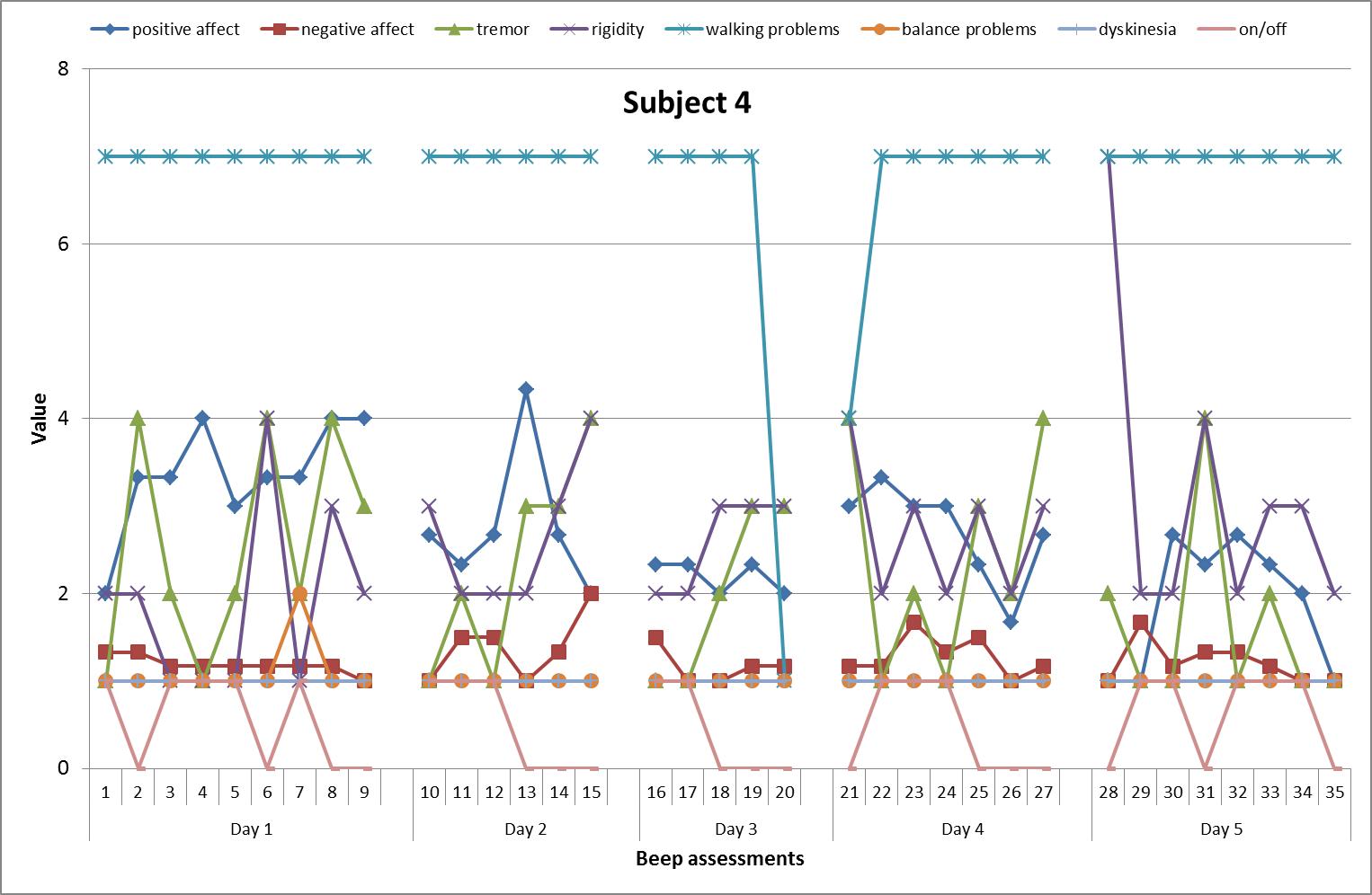


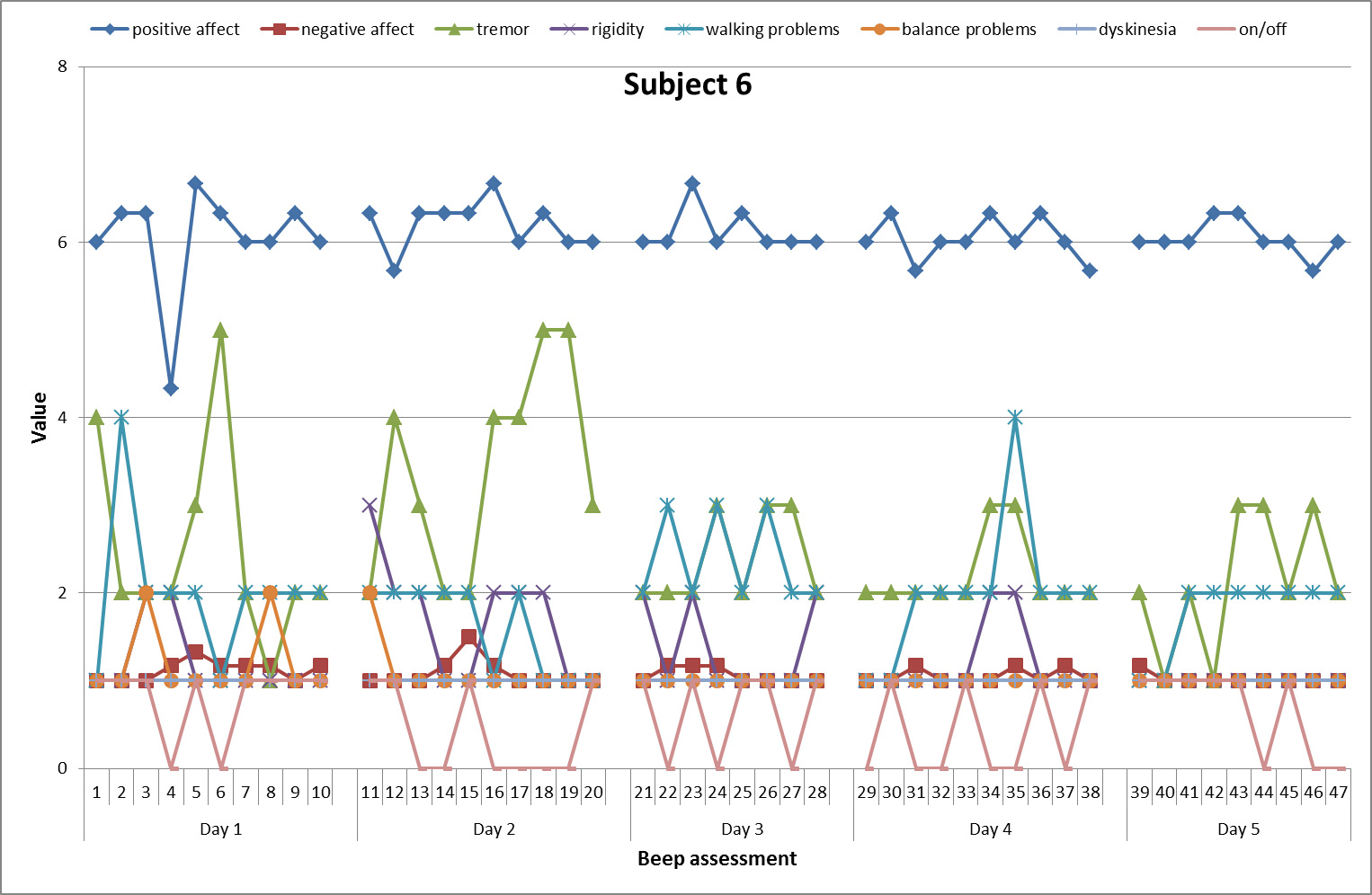

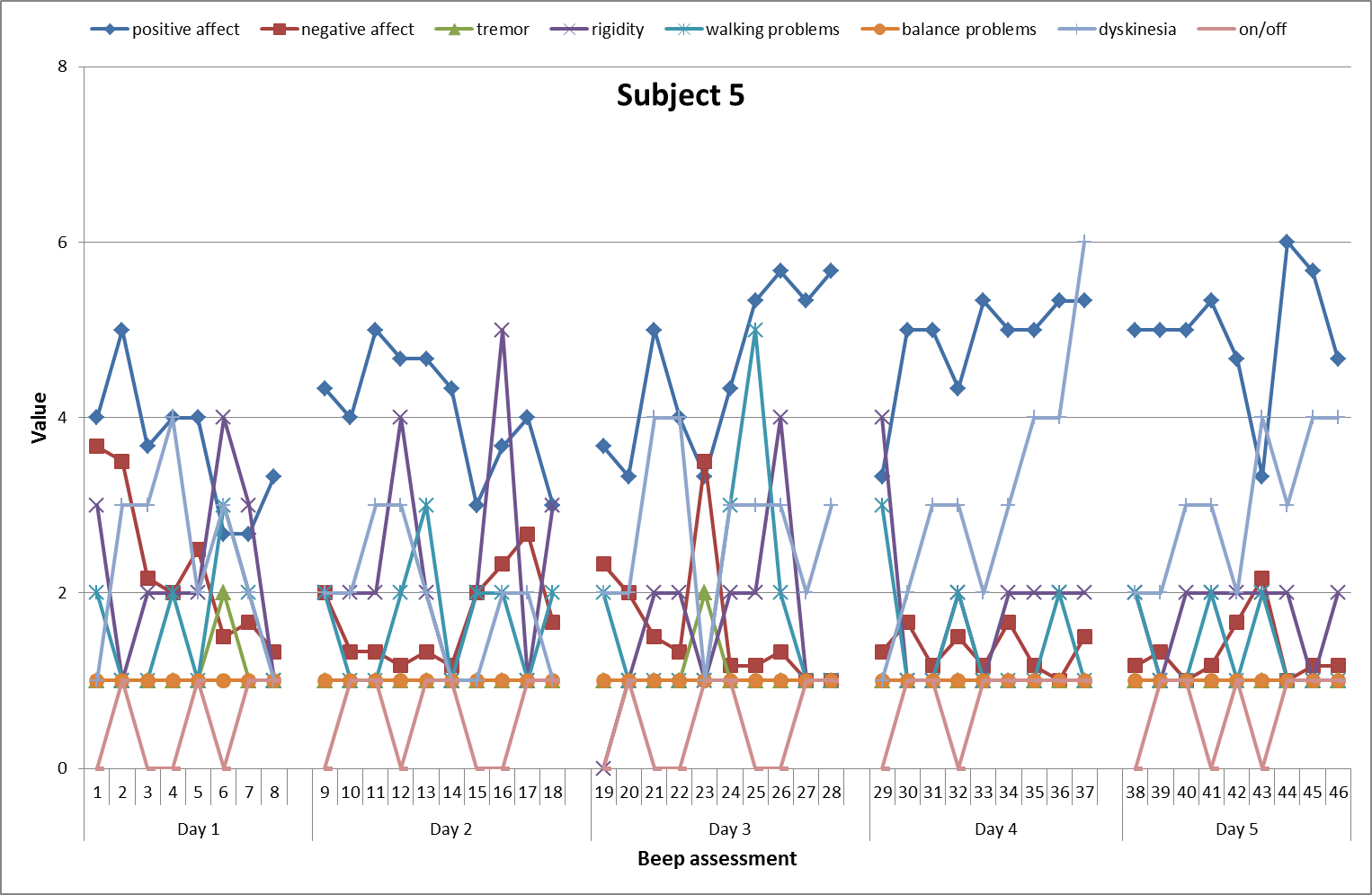


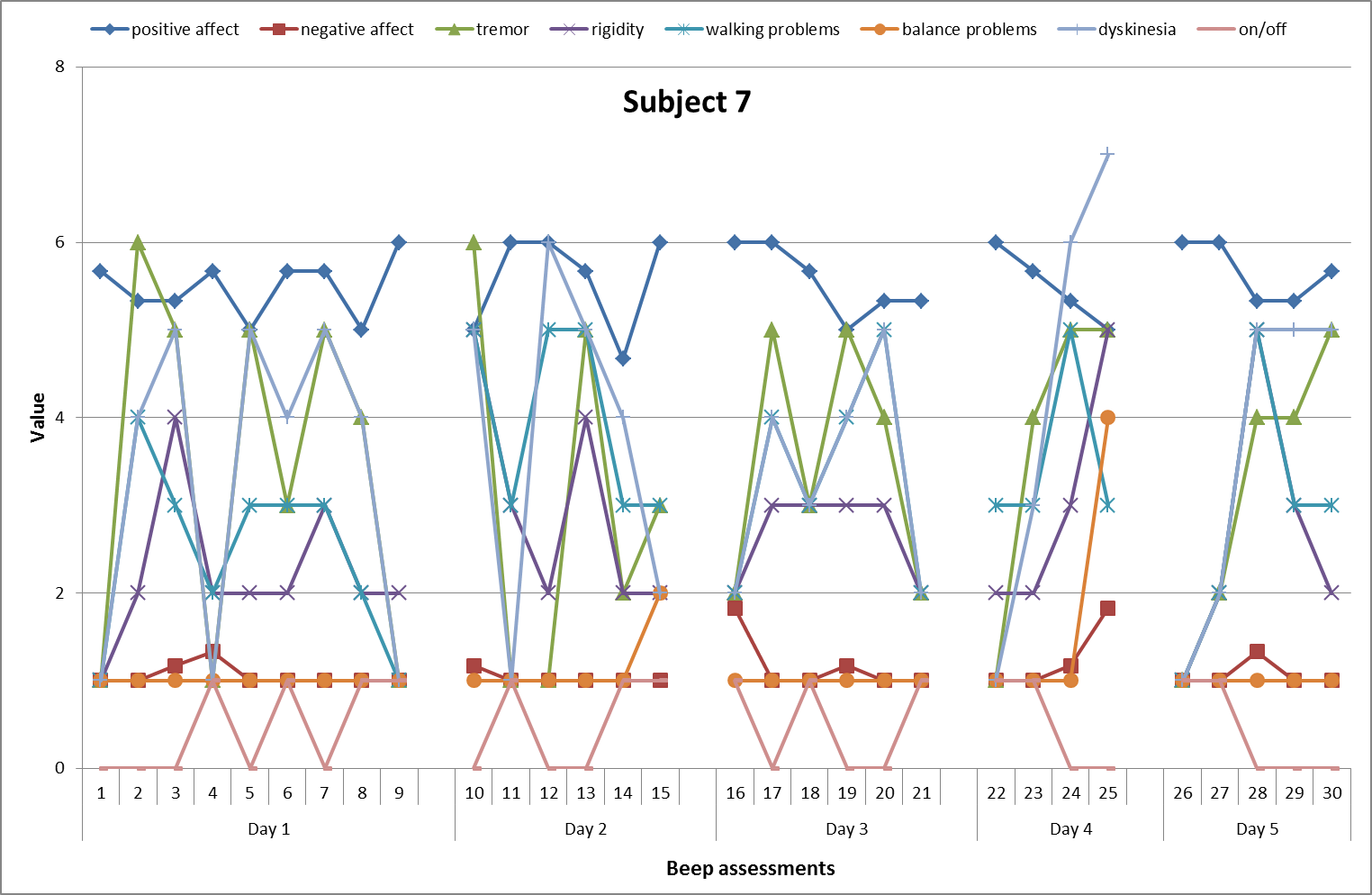


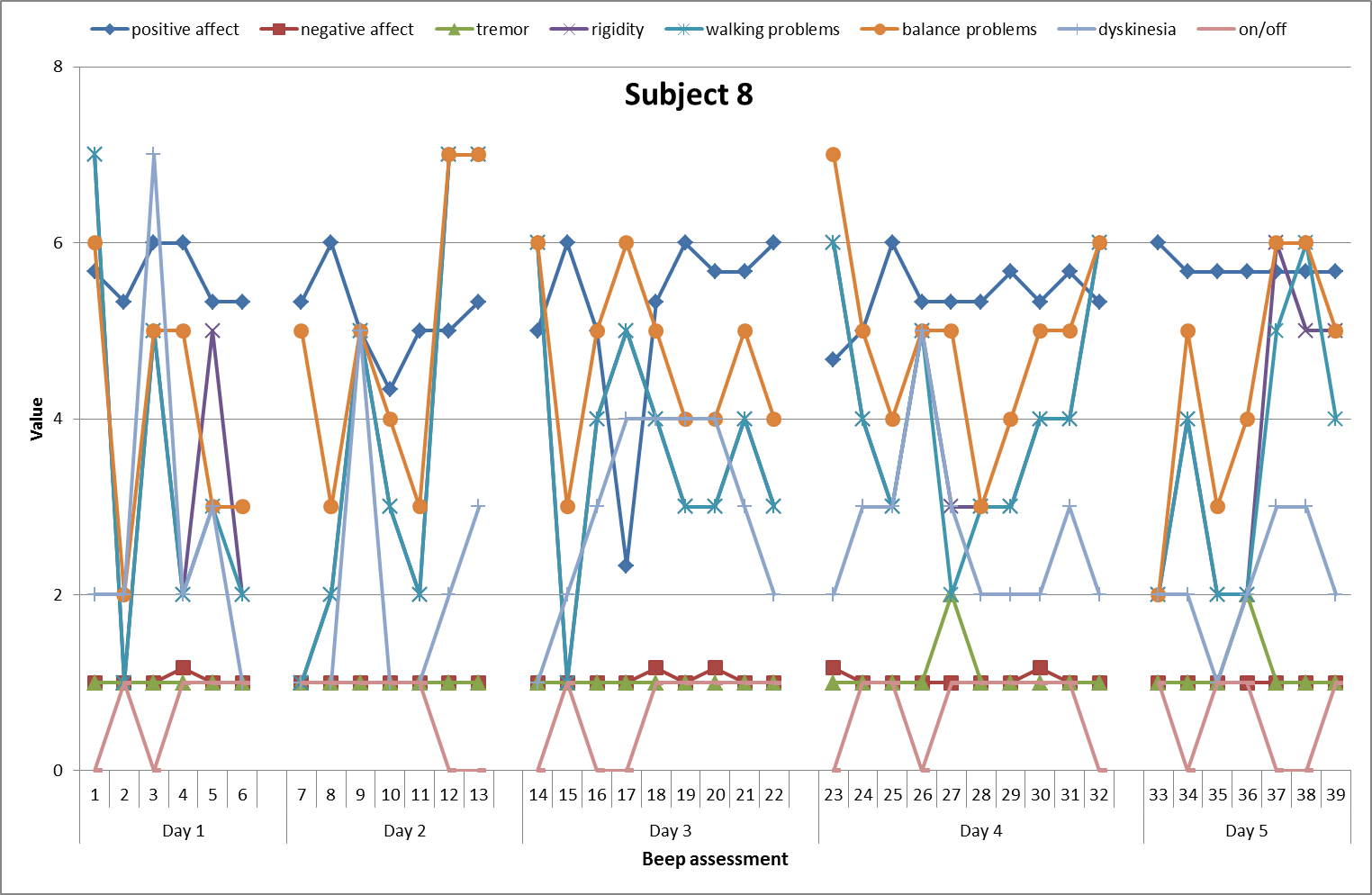


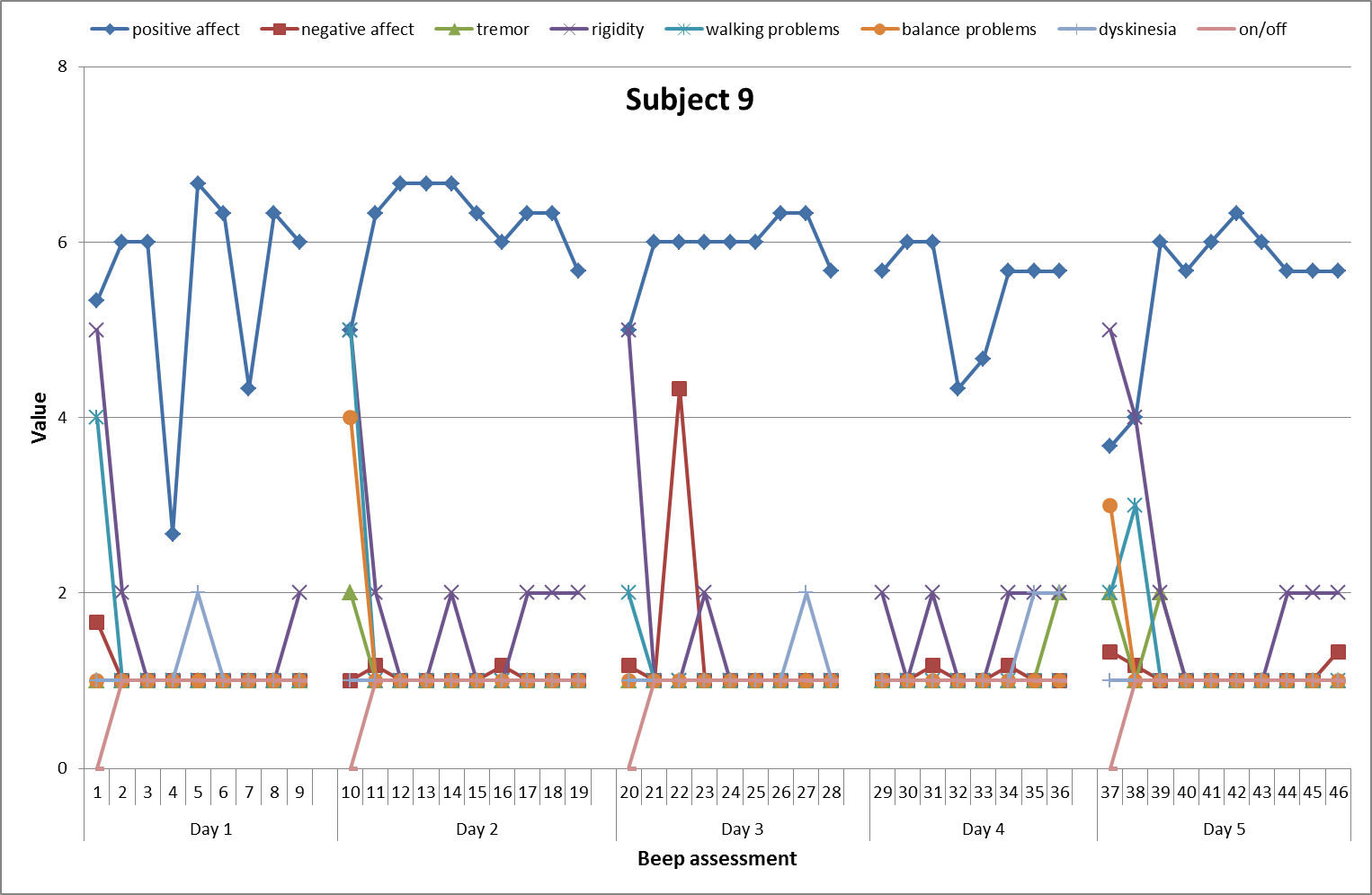

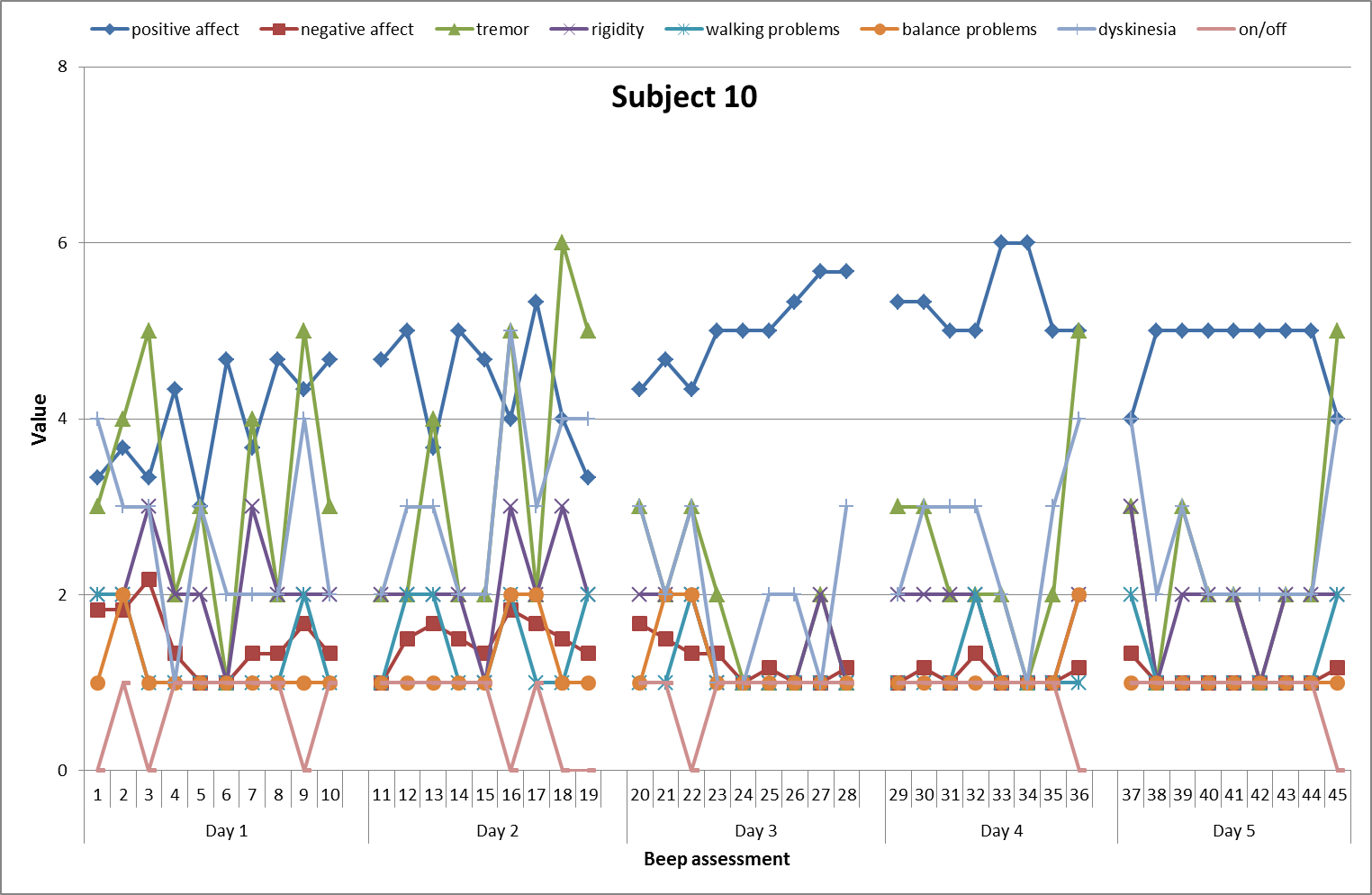


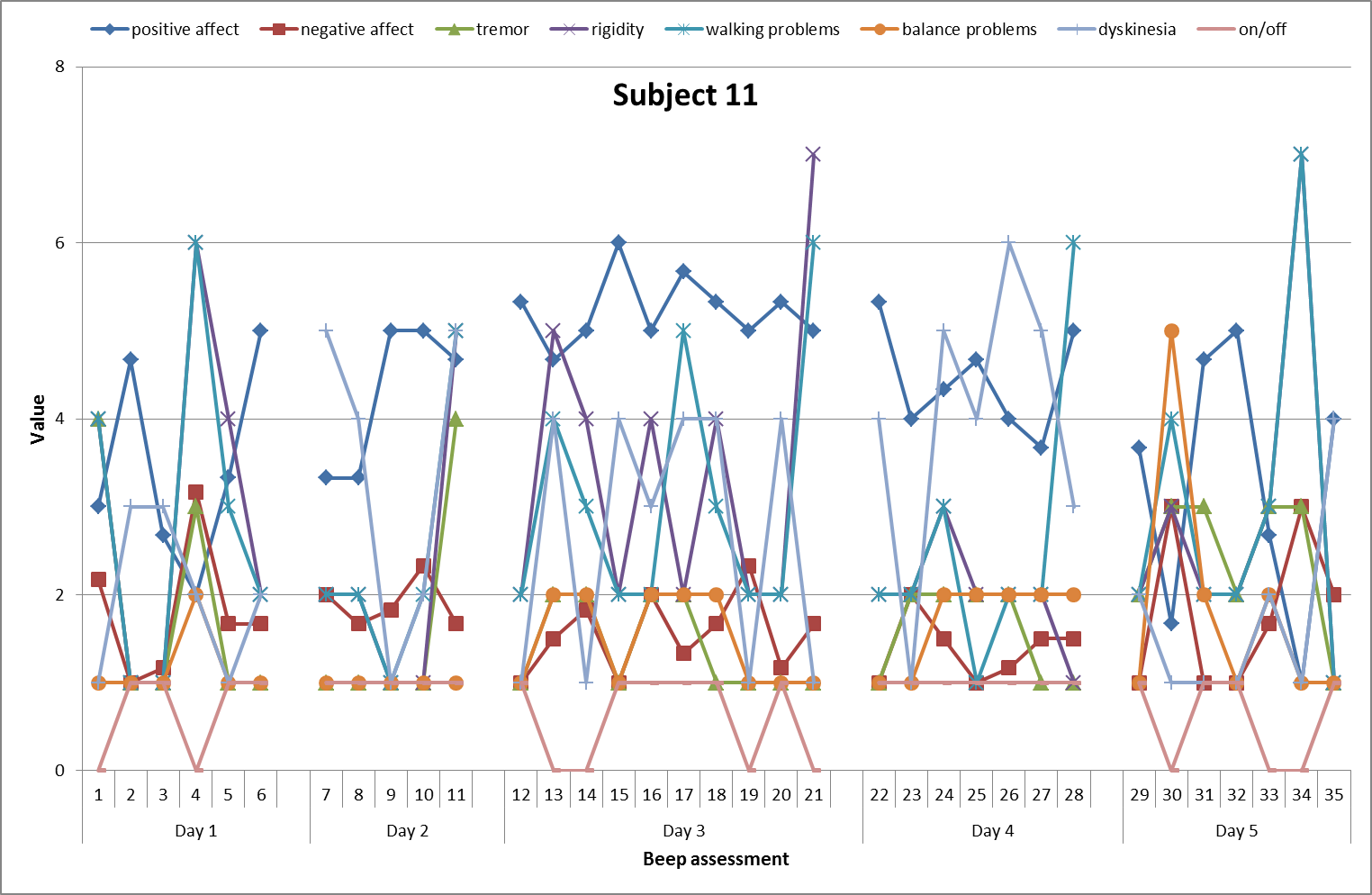

Supplement: Supplementary file 1 — Supplementary table 1: ESM questionnaire Supplementary table 2: Overview of response rates Supplementary table 3. Interaction effects ON/OFF state Supplementary table 4. P‐values original analyses and permutation analyses Supplementary table 5: Associations between motor symptoms and mood states in individual patients Supplementary table 6: Associations between motor symptoms and contextual factors in individual patients Supplementary figure 1: Moment‐to‐moment fluctuations in positive and negative affect and in tremor, rigidity, balance problems, walking problems and dyskinesia in individual patients [file MDS-35-1145-s001.docx]
